# The origin and effect of small RNA signaling in plants

**DOI:** 10.3389/fpls.2012.00179

**Published:** 2012-08-09

**Authors:** Jean-Sébastien Parent, Angel Emilio Martínez de Alba, Hervé Vaucheret

**Affiliations:** Institut Jean-Pierre Bourgin, UMR1318, INRAVersailles, France

**Keywords:** RNA silencing, miRNA, siRNA, cell-to-cell movement, systemic movement

## Abstract

Given their sessile condition, land plants need to integrate environmental cues rapidly and send signal throughout the organism to modify their metabolism accordingly. Small RNA (sRNA) molecules are among the messengers that plant cells use to carry such signals. These molecules originate from fold-back stem-loops transcribed from endogenous loci or from perfect double-stranded RNA produced through the action of RNA-dependent RNA polymerases. Once produced, sRNAs associate with Argonaute (AGO) and other proteins to form the RNA-induced silencing complex (RISC) that executes silencing of complementary RNA molecules. Depending on the nature of the RNA target and the AGO protein involved, RISC triggers either DNA methylation or chromatin modification (leading to transcriptional gene silencing, TGS) or RNA cleavage or translational inhibition (leading to post-transcriptional gene silencing, PTGS). In some cases, sRNAs move to neighboring cells and/or to the vascular tissues for long-distance trafficking. Many genes are involved in the biogenesis of sRNAs and recent studies have shown that both their origin and their protein partners have great influence on their activity and range. Here we summarize the work done to uncover the mode of action of the different classes of sRNA with special emphasis on their movement and how plants can take advantage of their mobility. We also review the various genetic requirements needed for production, movement and perception of the silencing signal.

## Introduction

**Figure 1:**
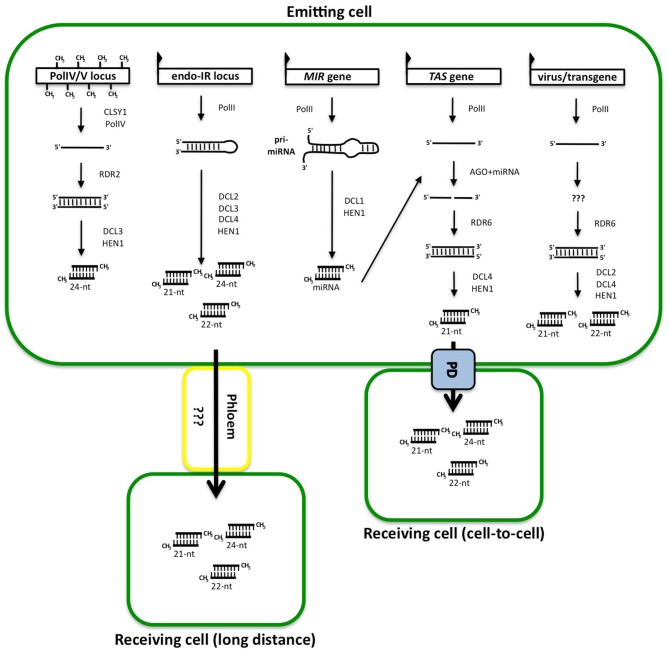
**Cascades of reactions leading to the production of sRNA duplexes, hypothetical constituent of the gene silencing signal.** The first RNA molecule is produced by RNA polymerases either from a silent locus (PolIV/V locus), an endogenous inverted-repeat (endo-IR locus), a miRNA (*MIR*) gene, a *TAS* gene or an integrated virus or transgene. The molecules are either folding back on themselves or made into dsRNA by RDR proteins after a known (in the case of *TAS* genes) or unknown (in the case of sense transgene) trigger. The dsRNA structure is then cut in one or several duplexes by DCL proteins and added methyl groups at the 5′ ends by HEN1. These duplexes are thought to be the signal and the effector of silencing when traveling from cell to cell through plasmodesmata (PD) or to long distance through the phloem.

Plants produce miRNAs and siRNAs, but no piRNAs. The biogenesis pathways responsible for the production of miRNA and siRNA molecules share a few similarities. They all derive from double-stranded RNA (dsRNA) molecules that are cleaved into one or many small RNA (sRNA) duplexes by one of the four dicer-like enzymes (DCL) found in plants (Baulcombe, 2004) as illustrated in the Figure 1. Most miRNAs are produced as single duplexes excised from short fold back stem-loops by DCL1 (Voinnet, 2009), although some young miRNAs are part of a series of duplexes sequentially processed from long fold back stem-loops by DCL4 (Rajagopalan et al., 2006). In contrast, siRNAs always come in populations of duplexes, which are processed from various types of precursors by DCL2, DCL3, and/or DCL4. siRNA precursors include near-perfect dsRNA molecule resulting from the fold-back of an inverted-repeat (IR) transcripts (Kasschau et al., 2007), or perfect dsRNA resulting from overlapping convergent transcription (Borsani et al., 2005), or transformation of single-stranded RNA into dsRNA by a RNA-dependent RNA polymerase (RDR). The resulting dsRNA molecules are generally cleaved sequentially into 21-, 22-, and 24-nt siRNAs by DCL4, DCL2, and DCL3, respectively.

Most of the sRNA duplexes are methylated at their 3′ extremities by the methyl-transferase HUA ENHANCER 1 (HEN1) (illustrated in Figure 1) to protect them from degradation (Li et al., 2005). One strand of the sRNA duplex is loaded on one of the Argonaute (AGO) proteins to form the core of the RNA-induced silencing complex (RISC). The identity of the AGO protein is determinant for the ultimate activity of the complex (Vaucheret, 2008). Classically, plant miRNAs and 21-nt siRNAs, which are produced by DCL1 and DCL4, associate with AGO1, AGO2, AGO7, or AGO10. Once they are associated to RISC, they cause post-transcriptional gene silencing (PTGS) of RNA messengers with near-perfect complementary sequence by translation inhibition (Brodersen et al., 2008) or slicing (Baumberger and Baulcombe, 2005). In contrast, the 24-nt siRNAs, which are produced by DCL3, associate with AGO4, AGO6, or AGO9 to trigger transcriptional gene silencing (TGS) (Brosnan and Voinnet, 2011). The enzyme DCL2 is responsible for the production of 22-nt siRNAs that are thought to act as backup for the 21-nt or the 24-nt siRNAs (Gasciolli et al., 2005), although in some cases, DCL2 acts antagonistically to the other DCLs (Bouché et al., 2006).

## Roles of sRNA in plants

### Targets of silencing

RNA silencing was discovered in plants as a mechanism whereby invading nucleic acids such as transgenes and viruses are silenced through the action of small homologous RNA molecules (Ding and Voinnet, 2007). It was later realized that RNA silencing also plays important roles in the regulation of endogenous gene expression in a much wider range of organisms (Carthew and Sontheimer, 2009).

#### Transgene silencing

Transgene reliable expression has been one of the major challenges of plant molecular biology, and RNA silencing has been one of its major obstacles. One of the first examples of PTGS was encountered while trying to over-express *chalcone synthase-A* (*CHS-A*) in petunia. Unexpectedly, cosuppression of endogenous and transgenic *CHS-A* was observed, resulting in a loss of flower pigmentation instead of an increase (Napoli et al., 1990). Subsequently, a growing number of reports have revealed the extent of transgene silencing phenomena and the diversity of silencing mechanisms. For instance, it was shown that TGS and PTGS can be achieved by transgenes producing hairpin RNA homologous to promoter or transcribed regions, respectively (Sijen et al., 2001). In contrast to IR transgenes, the overall scheme by which sense transgenes generate dsRNA remains elusive (Figure 1) (Beclin et al., 2002). Nevertheless, both sense and IR transgene-based systems have been instrumental to decipher silencing mechanisms through the identification of mutants and the characterization of the corresponding genes. These studies revealed a plethora of genes involved in sRNA biogenesis and allowed the dissection of their mode of action.

#### Resistance to pathogens

Expressing part of a viral genome into plants sometimes lead to virus resistance. The underlying mechanism was revealed when plants were shown to produce siRNAs corresponding to the infecting virus (Hamilton and Baulcombe, 1999). It is now known that plant posses an antiviral defense mechanism that is very similar to the mechanism by which sense transgenes are silenced by PTGS (virus/transgene in Figure 1). To summarize, transgene or viral RNA are somehow transformed into dsRNA and diced into primary siRNAs, which initiate silencing locally. siRNA-guided cleavage of the RNA target(s) initiates the production of secondary siRNAs through the sequential action of RDRs and DCLs (Wang et al., 2011). Then, antiviral response spreads systemically throughout the plant to promote resistance. Consistently, mutants defective in sense transgene-triggered PTGS exhibit hypersusceptibility to virus infection [reviewed in Ding and Voinnet (2007)].

In addition to plant antiviral defense responses that rely on siRNAs derived directly from the genome of the pathogen, there are now indications that host-, as opposed to parasite-encoded sRNAs might also participate in antiviral defense. Indeed, two miRNAs, bra-miR158, and bra-miR1885, are significantly upregulated during *Brassica rapa* infection by Turnip mosaic virus (TuMV) (He et al., 2008). However, this response appears highly specific to TuMV infection because similar experiments performed on *B. rapa* and *Brassica napus* with Cucumber mosaic virus, Tobacco mosaic virus, or the fungal pathogen *Sclerotinia sclerotiorum* showed no induction of either miRNA. Interestingly, the predicted target for bra-miR1885 is a member of the TIR-NUCLEOTIDE-BINDING SITE DOMAINS (NBS)-C-terminal LEUCINE-RICH REPEATS (LRR) class of disease-resistant proteins. It is therefore possible that the reported induction of the miRNA reflects an attempt from the pathogen to use an endogenous plant system to lower its defenses rather than a *bona fide* plant defense response.

Genome-encoded miRNAs have also been shown to contribute to resistance against bacteria. One notable example is miR393, which, in Arabidopsis, is induced by treatment with the Flg22 peptide, derived from the bacterial flagellin, a well-known pathogen-associated molecular pattern mimicking bacterial infection (Navarro et al., 2006). miR393 is an endogenous regulator of auxin signaling, which targets *TIR1, AFB2*, and *AFB3* controling auxin response (Dharmasiri et al., 2005). During infection, plants must downregulate their development to allocate a maximum of resources towards pathogen resistance (Navarro et al., 2006). miRNAs therefore represent a mean to rapidly shut down auxin-mediated growth. Conversely, overexpression of miR393a from a strong constitutive promoter results in lower levels of *TIR1* mRNA and restricted bacterial growth. One can expect that many more examples will be put in light as our knowledge on sRNA-mediated processes deepens.

#### Regulation of developmental genes

Although a large portion of the plant genome is actively transcribed into RNA, only a small fraction encodes proteins. In many cases, non-protein coding RNAs produce sRNAs which direct either transcriptional or post-transcriptional repression of genes with conserved cellular functions and serve as a flexible sequence-specific source of regulation that promotes adaptability (Dunoyer et al., 2010a). Behind 24-nt siRNAs from endogenous TGS, miRNAs are the second most abundant class of sRNAs in plants and the majority of them are predicted to target genes involved in several aspects of development, including meristem division, organ separation, leaf shape, secondary root elongation, flowering time, fertility, etc. (Voinnet, 2009).

The importance of miRNAs is illustrated by the fact that several miRNAs regulates the functioning of the miRNA pathway. Indeed, feedback loops control the expression of *DCL1* and *AGO1* genes, which are essential for miRNA biogenesis and activity, respectively. *DCL1* is a target of miR162, which target the cleavage of *DCL1* mRNA (Xie et al., 2003). *AGO1* expression is tightly regulated in both an AGO1/AGO10-dependent manner by negative feedback loops involving miR168 and *AGO1*-derived siRNAs (Vaucheret et al., 2004, 2006; Mallory and Vaucheret, 2009).

Plants have further adapted RNA silencing to regulate protein-coding genes through a class of siRNA known as *trans*-acting siRNAs (ta-siRNAs) (Peragine et al., 2004; Vazquez et al., 2004; Allen et al., 2005). ta-siRNAs are endogenous siRNAs that, like miRNAs, regulate genes different from those from which they originate and thus act *in trans*. In Arabidopsis, ta-siRNAs are produced by two mechanisms: capped and polyadenylated transcripts from *TAS1, TAS2*, and *TAS4* loci are channeled into the ta-siRNA pathway by a cleavage event triggered by 22-nt miRNA (miR173 and miR828) associated with AGO1, whereas capped and polyadenylated transcripts from the *TAS3* locus are cleaved by miR390 associated with AGO7 (Figure 1). Both associations differ from the classical 21-nt miRNA-AGO1 association in that the cleaved *TAS* transcripts are copied into dsRNA by RDR6 and converted to siRNAs by DCL4. Interestingly, while a single miRNA target site is found in *TAS1a, TAS1b, TAS1c, TAS2*, and *TAS4* sequences (Rajagopalan et al., 2006), two miRNA target sites are found in *TAS3* (Allen et al., 2005). Although one of these two miRNA sites does not involve slicing, it restricts the production of ta-siRNAs to the region comprised between the two miRNA (Axtell et al., 2006). In the case of *TAS1a, TAS1b, TAS1c*, and *TAS2*, ta-siRNAs are primarily produced from the region comprised between the unique miRNA site and the poly-A tail, and then restricted to a shorter region defined by the miRNA site and a second cleavage site corresponding to a *TAS1c*-derived ta-siRNA (Rajeswaran and Pooggin, 2012), The resulting ta-siRNAs regulate their target mRNAs in the same manner as miRNAs do, i.e., in association with AGO1 (Howell et al., 2007).

#### Transposon taming

Plant genomes contain massively abundant and unstable transposable elements (TE), most of which are inactivated or silent because of epigenetic suppression (Wang et al., 2009; He et al., 2010). The sole purpose of TEs is to increase their copy number, which imposes a constant threat to the integrity of the host genome. Therefore, inactivation of TEs is pivotal for the survival of the host, and sRNAs contribute to TE silencing through two different pathways. In the major pathway, 24-nt siRNAs derived from the transposons through PolIV/V transcription trigger DNA methylation and chromatin modification, which likely suppresses PolII transcription of TEs. Given the abundance of TEs in plant genomes, it is not surprising to find that 24-nt is the most abundant class of sRNAs (Kasschau et al., 2007). An alternative pathway involves 21-nt siRNAs derived from the transposons, which likely trigger the degradation of TE transcripts by PTGS. This pathway operates in particular cells where the 24-nt siRNA pathway is inactive (Slotkin et al., 2009).

On the other hand, TEs are an important source of epigenetic novelty. Indeed, some of the genetic changes caused by TEs, including alterations in gene expression, gene deletion and insertion, and chromosome rearrangements, can be beneficial at the population scale. This requires the movement of TEs to new positions within the host genome under certains circumstances, which requires escaping from silencing by sRNAs. Supporting this model, Arabidopsis plants impaired in 24-nt siRNA production showed stress-dependent activation of retrotransposons in germinal cells (Ito et al., 2011). Recently, it has been shown that TEs can be activated at specific time and in determined spaces such as cell lineages that are adjacent to the germ line. Due to down-regulation of the silencing machinery in pollen vegetative nuclei, TEs are activated and their transcription is used to generate siRNAs that reinforce silencing in the germinal nuclei (Slotkin et al., 2009). Similarly, at the transition between juvenile and reproductive stages, a key component of the ta-siRNA silencing pathway is down-regulated in leaves. This results in the upregulation of a target of this pathway and the loss of transcriptional silencing of a TE, suggesting a link between a commitment to reproductive competence and TE silencing (Li et al., 2010).

### Evidences for silencing movement

Early on, grafting experiments have showed that PTGS can propagate to different parts of the plant (Palauqui et al., 1997; Voinnet et al., 1998). Additionally, short-distance propagation was observed when the expression of dsRNA was restricted to certain cell types (Himber et al., 2003; Dunoyer et al., 2005; Smith et al., 2007). More recently, TGS was also shown to move at long distance (Brosnan et al., 2007). The corollary from these observations is that a silencing signal must move from cell to cell in the form of either an active sRNA or a precursor molecule. Although more experiments are required to determine precisely the RNA transport mechanism, it appears likely that the 21-, 22-, and 24-nt molecules themselves are the main carriers of the silencing signal (Brosnan and Voinnet, 2011).

## Movement of sRNA molecules

### Intracellular movement

Within cells, RNA silencing can spread from an inducing locus to homologous targets present at unlinked loci, a phenomenon generally referred to as *trans*-silencing or homology-dependent gene silencing. This phenomenon allows the taming of ectopic copies that result from gene duplication, TE movement or plant transformation, and which could lack the appropriate sRNA-mediated regulation. For example, 24-nt siRNAs produced from the promoter of the endogenous *FLOWERING WAGENINGEN* (*FWA*) gene trigger *trans*-TGS on ectopic *FWA* copies introduced by transformation (Greenberg et al., 2011). Similarly, 21-nt siRNAs produced from the transcribed region of gene undergoing PTGS trigger *trans*-PTGS on unlinked genes expressing homologous RNAs. When targeted to transgenes, *trans*-PTGS often spreads *in cis*, outside of the region of homology but still within the transcribed region of the target, a phenomenon generally referred to as transitivity. This was well illustrated by the use of a viral trigger or an IR transgene containing only the GF part of the Green Fluorescent Protein (*GFP*) coding sequence (Vaistij et al., 2002). The introduction of this construct into a transgenic plant constitutively expressing *GFP* under the control of a viral promoter led to *GFP* PTGS and the production of secondary 21-nt siRNAs from the whole *GFP* gene, including the P part that is missing from the inducer of silencing. This is thought to happen consequently to the slicing of a first mRNA molecule by an AGO protein and depends on RDR6 to synthesize complementary RNA using the primal RNA cleavage product as template. Similarly, spreading and production of secondary 24-nt siRNAs was observed at promoter regions during *trans*-TGS (Daxinger et al., 2009; Melnyk et al., 2011a). In this case, transitivity involves RDR2.

Another important form of intracellular movement occurs between the nucleus and the cytoplasm. Indeed, it was assumed that part of the silencing machinery (associated with TGS) is restricted to the nucleus while other factors (associated with PTGS) remain in the cytoplasm. This has important consequences for the silencing models; for instance, it was long thought that the PTGS machinery was localized exclusively in the cytoplasm, forbidding that sRNA could be generated against a pre-mRNA. However, a recent study has showed that an intron introduced in plants as an IR could cause the silencing of the corresponding gene (Hoffer et al., 2011). Moreover, the presence of both RDR6 and DCL4 in the nucleus strongly suggests that some part of the PTGS reaction is occurring in the nucleus. This could also explain why nuclear TGS-associated factors such as CLSY1, RDR2, NRPD1a, and JMJ14 are required for 21-nt-associated PTGS mediated by IR transgenes (Dunoyer et al., 2007; Smith et al., 2007; Searle et al., 2010). Conversely, it was recently reported that the majority of the 24-nt population associated with TGS are in fact present in cytoplasm and not in the nucleus (Ye et al., 2012). These sRNAs would therefore have to be shuttled through the nuclear membrane to be effective. For now, only the Arabidopsis HASTY factor has been proposed to be involved in the shuttling of PTGS molecules between the two compartments (Park et al., 2005). Careful study of the localization of the various silencing factors will be required to better understand the subtleties of the silencing mechanisms.

### Cell-to-cell movement

Early studies of transgene-mediated PTGS have demonstrated that RNA silencing is able to spread to about 10–15 cells away from where it was initiated (Himber et al., 2003). This silencing route appears to be distinct from the systemic silencing signal as demonstrated by the use of a modified virus to silence a constitutive *GFP* transgene (Ryabov et al., 2004). Today, it is mostly accepted that RNA molecules carry the signal from cell to cell through the plasmodesmata (Yoo et al., 2004) although this model is based on mostly indirect observations (Kobayashi and Zambryski, 2007). Studies performed with artificial miRNAs and ta-siRNAs revealed that, on average, ta-siRNAs have greater action ranges than miRNAs (De Felippes et al., 2011). This type of short-range signaling offers some interesting possibilities to the organism. For instance, since the cell-to-cell silencing signal is not amplified, it is assumed that the concentration of the corresponding sRNA is diluted as it moves away from the emitting cell (Himber et al., 2003). This type of gradient can be used for dose-dependent regulation of cell fate, much like morphogens in animals (Skopelitis et al., 2011). A good example is miR165/166, which is produced in root endodermal cells and diffuses to establish an inverse gradient of the PHB transcription factor that determines the cortex, pericycle, protoxylem and metaxylem cell fates (Carlsbecker et al., 2010). Cell-to-cell movement of siRNAs could also serve to transfer epigenetic information from the vegetative cell to the germinal cells of developing pollen grains (Slotkin et al., 2009) but this is likely to involve a different transport mechanism because these cells are not known to possess plasmodesmata. Also, the fact that a silencing signal from a host plant can trigger down-regulation of the corresponding gene in a fungal pathogen argues that the RNA molecules can be exchanged without the plasmodesmatal connection (Nowara et al., 2010).

### Systemic movement

Even before the discovery of sRNAs, it became obvious that whatever the signaling molecule was, silencing was able to move throughout the plant (Palauqui et al., 1997; Voinnet et al., 1998). The source-to-sink profile of this movement and the presence of RNA molecules in the phloem sap (Yoo et al., 2004) indicate that the signal likely moves through the phloem rather than the xylem. Recently, it has been proven that about 35% of endogenous siRNA-generating loci in Arabidopsis are associated with long-range mobility highlighting the importance of this phenomenon (Molnar et al., 2010). Micro-grafting experiments have been most indicative in the attempt to understand how this message is carried (Brosnan et al., 2007; Dunoyer et al., 2010b; Molnar et al., 2010). They have revealed that, in Arabidopsis, siRNAs of all sizes can cross the graft junction although in absolute terms, the 24-nt are the most abundant ones (Molnar et al., 2010). Because the 24-nt siRNAs are associated with RNA-directed DNA methylation, these observations raised the hypothesis that a TGS signal could travel through the plant to reach the meristem where it could cause heritable epimutations (Brosnan and Voinnet, 2011).

The ability of riboregulators to traffic throughout the plant provides obvious advantages to the organism. First, this signal could coordinate the adaptation to an environmental cue in the entire plant after the cue is perceived by a few cells. For example, regulating the intake of phosphate involves miR399, which is present in the phloem sap and moves from shoot to root where it downregulates its target (Pant et al., 2008). Another clear advantage is the systemic resistance to viruses. Indeed, a single cell that has been infected by a virus could send virus-derived siRNAs to the neighbor cells and also to distant organs before the viral RNA ever reached them. Importantly, long-distance siRNA movement could reach the meristems, thus excluding most viruses from these important cells (Schwach et al., 2005). Recently, it was even proposed that fast-evolving IR loci producing endogenous siRNAs are in fact environmental sensors allowing a single plant to adapt to a new environmental condition and transmit epigenetic information to its progeny (Dunoyer et al., 2010a). Although this hypothesis is attractive, it remains to be proved that such an adaptation is possible and then inheritable.

## Nature of the sRNA signal and genetic requirements

### Nature of the signal

The exact form of the silencing signal(s) is still under debate. Obviously, it must contain nucleic acids, most certainly RNA, to ensure sequence-specificity. However, whether it is a long or short, single- or double-stranded molecule remains unclear. Moreover, it is possible that different forms of silencing signals exist (Melnyk et al., 2011b). There is also a question as to how far signaling molecules can travel and if cell-to-cell movement and long-distance signaling are two sides of the same mechanism (Brosnan and Voinnet, 2011).

To address some of these questions, the movement of fluorescent 21- and 25-nt sRNAs was tested by microinjection in *Nicotiana benthamiana* leaves (Yoo et al., 2004). Surprisingly, these molecules did not show any movement outside of the injection area. It is possible that the microinjection technique prevents the movement of sRNA or that the sRNA molecules produced by Yoo et al. were chemically unfit for movement. In a more recent study, Dunoyer et al. bombarded different forms of *GFP*-derived siRNA molecules into transgenic Arabidopsis leaves (Dunoyer et al., 2010b). They first showed that 21-nt duplexes could move beyond the bombarded site and caused silencing of a *GFP* in surrounding cells. Using a similar approach, they then showed that 24-nt duplexes also move outside of the bombarded cell (Dunoyer et al., 2010a). Although these studies proved that siRNAs move, they do not preclude that other forms of RNA molecules could also carry the silencing signal (Melnyk et al., 2011b).

Another approach used micro-grafting of Arabidopsis plants followed by sRNA deep sequencing. These experiments confirmed that both primary and secondary siRNAs of all sizes are able to move through the graft union (Melnyk et al., 2011a). This result is in line with the hypothesis that mature siRNAs are moving. Indeed, a *dcl2dcl3dcl4* rootstock, which is unable to process dsRNA into siRNAs, was still able to receive the silencing signal when grafted to wild-type trigger-expressing shoots, confirming that, at least, siRNAs are moving. However, a more thorough examination of the content of the phloem sap might be required to determine with certainty what type of molecules are indeed moving.

### Genetic requirements for the production of the silencing signal

With the exception of a few miRNAs that are produced in companion cells and loaded into the phloem and also miR165/166 and miR390 that have been proposed to move at short distance (Carlsbecker et al., 2010; Marin et al., 2010), miRNAs generally act in a cell-autonomous manner. In contrast, there is clear evidence for 24-nt siRNA long-distance mobility and for greater short-distance mobility of 21-nt siRNAs compared with miRNAs (Molnar et al., 2010; De Felippes et al., 2011). Because miRNAs and siRNAs are quite similar, these differences in mobility must be related to their biogenesis mode and/or AGO partners. Indeed, as illustrated in Figure 1, miRNA duplexes are processed by DCL1 from short imperfect foldback stem-loops, whereas siRNA duplexes are processed by DCL2, DCL3, and/or DCL4 from longer and near perfect dsRNA molecules such as long inverted repeats, antisense transcript pairs or RDR products. Consistent with their essential roles in the production of 21- and 24-nt siRNAs, DCL4 was shown to be of paramount importance for generating a short distance PTGS signal (Dunoyer et al., 2010b), whereas DCL3 was required for generating a long-distance TGS signal (Melnyk et al., 2011b). Indeed, restricted expression of *DCL4* to companion cells was sufficient to restore short distance spreading of PTGS to adjacent cells in a *dcl4* mutant (Dunoyer et al., 2010b). Moreover, grafting of *dcl3* shoots containing the trigger for TGS onto WT roots prevented the transmission of silencing (Melnyk et al., 2011a).

Given that most miRNAs and 21-nt siRNAs bind to AGO1 after duplex separation, it is tempting to speculate that the difference in mobility between miRNAs and 21-nt siRNAs is related to the DCL involved in their biogenesis, rather than the AGO to which they associate. However, association with other AGOs than AGO1 may also contribute to mobility. Indeed, miR165/166 and miR390, which were shown to move at short distance, associate with AGO10 and AGO7, respectively (Montgomery et al., 2008; Zhu et al., 2011). Moreover, 24-nt siRNAs, which move more efficiently at long distance than 21-nt siRNAs, associate with AGO4, AGO6, and AGO9, instead of AGO1 (Havecker et al., 2010). Therefore, the entire channeling pathway of a given sRNA may contribute to its degree of mobility.

### Genetic requirements for the traveling of the silencing signal

It is surprising that no factor involved in the actual movement of silencing-associated molecules has been identified through forward genetic screens yet (Melnyk et al., 2011b). Indeed, some experiments were specifically designed to identify these factors and only turned up genes potentially involved in either the production or the perception of silencing (Dunoyer et al., 2005; Smith et al., 2007). Although these screens do not allow the spatial dissection (origin, movement or reception) of the silencing signal, it does not seem like any actor identified so far is directly involved in the movement of the RNA molecules (Melnyk et al., 2011b). A few reasons may explain these observations; first and simplest, the factors involved in movement may be required for development and/or fertility of the plant, preventing the isolation of a viable mutant. Alternatively, many proteins may have redundant function in this pathway and would therefore escape the forward screen. It will be interesting in the future to see if other systems will make up for these shortcomings or if reverse genetic screens will be informative in this regard. Interestingly, a recombinant RNA-binding protein from *Cucurbita maxima* was shown to increase cell-to-cell movement of exogenous sRNAs when micro-injected in *N. benthamiana* (Yoo et al., 2004). This last result suggests that binding partners, other that AGO proteins, may be involved in the silencing movement between cells. Moreover, recent evidences indicate that AGO proteins participate to the shuttling of the sRNA molecules between the cytoplasm and the nucleus (Ye et al., 2012).

### Genetic requirements for the perception of the silencing signal

In the hope of revealing the factors involved in sRNA silencing movement, two similar approaches have been used. They involved the companion cell-specific expression of an IR triggering the silencing of an endogenous gene of Arabidopsis, either the *SULFUR* (*SUL*) (Dunoyer et al., 2005) or *PHYTOENE DESATURASE* (*PDS*) genes (Smith et al., 2007). In these plants, the siRNAs derived from an IR construct are produced in the companion cells and the silencing of the endogenous targets 10–15 cells located around the veins causes localized photo-bleaching. Genetic screens using this system have revealed mutations that were able to either abolish or enhance the movement of silencing [reviewed in Melnyk et al. (2011b)]. Surprisingly, the results of these screens show that the nuclear NRPD1 and NRPD2 (the largest subunits of PolIV), RDR2, CLSY1, and JMJ14 factors are involved in the reception of the signal rather than its production. This was unexpected given that these factors usually play a role in TGS. As suggested previously, this may reveal interdependence between the TGS and PTGS pathways. Using the same system, Dunoyer et al. demonstrated the implication of AGO1 in the reception of the signal rather than production. Indeed, restricted expression of *AGO1* in the companion cells of an *ago1* mutant does not rescue the vein photo-bleaching phenotype associated with *SUL* silencing, indicating that AGO1 plays an essential role in the receiving cells (Dunoyer et al., 2010b). This contrasts *DCL4* for which expression in the companion cells of a *dcl4* mutant is sufficient to rescue the vein photo-bleaching phenotype.

The screens described above also confirmed that the 21-nt sRNAs are essential for the short distance propagation of PTGS. Indeed, the intensity of the photo-bleaching phenotype is directly correlated to the abundance of the 21-nt. It is therefore expected that any mutation influencing the abundance of these sRNAs would influence the overall phenotype. This is probably why mutations in *DCL3, AGO4, DRD1*, and *POLV* genes release partial TGS of *IR-PDS* inducing transgenes, thus increasing the amount of *PDS* 21-nt siRNAs and the photo-bleaching phenotype (Smith et al., 2007; Dong et al., 2011).

Lastly, it is interesting to note that silencing of endogenous genes does not propagate systemically in an RDR6-dependent manner, and remains localized to the 10–15 cells neighboring the veins. Indeed, mutations in *RDR6* did not affect local *PDS* PTGS caused by *IR-PDS* inducing transgenes expressed in companion cells (Himber et al., 2003). This result is consistent with RDR6 participating in the perception rather than the production of long distance PTGS signal (Schwach et al., 2005). However, it is interesting to note that RDR6 from *N. benthamiana* was shown to contribute to the virus-induced cell-to-cell silencing signal (Qin et al., 2012). It is therefore possible that RDR6 is involved in both systemic and local silencing signal but with a different level of contribution to each mechanism.

## Concluding remarks

Obviously, the movement of RNA silencing signals is crucial for plants. What appeared primarily as a simple and straightforward mean to counter-attack RNA viruses has turned into a complex battery of traveling molecules insuring the coordinated development of the organism as well as genome stability and perhaps even the capacity for fast adaption to the environment. With such important implications in the various biological functions of the plant, it is not surprising that RNA interference raises high interest in the fields of agriculture and biotechnology. Indeed, sRNAs now appear as a promising mean to be able to modify the plant metabolism in any number of ways. The potential practical applications of gene silencing emphasize the importance of answering the leftover questions regarding the sRNA molecules. For instance, it remains to be confirmed if sRNAs are actually the silencing effectors of these processes. Also, the carriers allowing movement through plasmodesmata and/or into the phloem remain to be identified. Increasing our knowledge in this field will also be of vital importance for the understanding of cell fate, defense mechanisms, stress adaptation and evolution of plants.

### Conflict of interest statement

The authors declare that the research was conducted in the absence of any commercial or financial relationships that could be construed as a potential conflict of interest.
